# The aortic valve with two leaflets

**DOI:** 10.1016/j.xjon.2021.09.050

**Published:** 2021-10-23

**Authors:** Justin T. Tretter, Diane E. Spicer, Jeffrey P. Jacobs, Robert H. Anderson

**Affiliations:** a Department of Pediatrics, Heart Institute, Cincinnati Children's Hospital Medical Center, University of Cincinnati College of Medicine, Cincinnati, Ohio; b Division of Cardiovascular Surgery, Departments of Surgery and Pediatrics, Congenital Heart Center, UF Health Shands Hospital, University of Florida, Gainesville, Fla; c Johns Hopkins All Children's Hospital, Johns Hopkins University, Saint Petersburg, Fla; d Cardiovascular Research Centre, Biosciences Institute, Newcastle University, Newcastle upon Tyne, United Kingdom

To the Editor:

**Figure:**
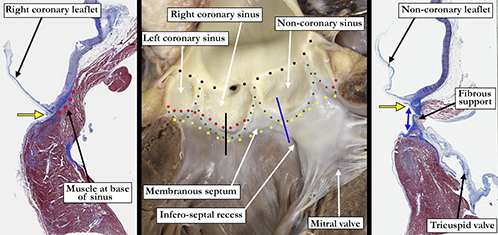

Dr Jacobs is a Consultant for SpecialtyCare and the American Academy of Dermatology. All other authors reported no conflicts of interest.

The *Journal* policy requires editors and reviewers to disclose conflicts of interest and to decline handling or reviewing manuscripts for which they may have a conflict of interest. The editors and reviewers of this article have no conflicts of interest.

We congratulate the authors of the recently published “International Consensus Statement on Nomenclature and Classification of the Congenital Bicuspid Aortic Valve and Its Aortopathy.”^1^ Any evidence-based consensus is to be applauded. The recent “international consensus” regarding the aortic valve is said to be subject to change in accordance with evidence-based data.^1^ As is also stated, “nomenclature refers to the choice of 'name' that is given to a particular structure, abnormality or phenotype.” For more than 2 decades, our International Society for Nomenclature of Paediatric and Congenital Heart Disease (https://ipccc.net/) has been seeking to produce such names for the various congenitally malformed hearts, including the bicuspid aortic valve.^2^

We also agree that classifications should be based on the English language. In this regard, “cusp” is currently used in a confusing fashion. A “Tower of Babel” was identified with regard to whether the moving parts of the aortic root should be described as “cusps” or “leaflets,” with “leaflet” being preferred.^3^ The International Consensus has chosen to use “cusp.” Had this been its only usage, there would have been no problem. In the legend to Figure 11, however, the coronary arteries are initially described as arising from the arterial valvar sinuses but then potentially arising “from each cusp.”^1^ “Cusp” is often used indiscriminately to account for both leaflets and sinuses. The better option is to be descriptive, describing leaflets and sinuses, particularly for those aiming surgically to preserve the dysfunctional valve.

The interleaflet triangles, given little attention in this consensus, are described as being “inter-cusp.”^1^ The extent of their development, or lack thereof, underlies the spectrum of abnormality.^4^ Their understanding serves to guide surgical repair.^5^

**Figure 1:**
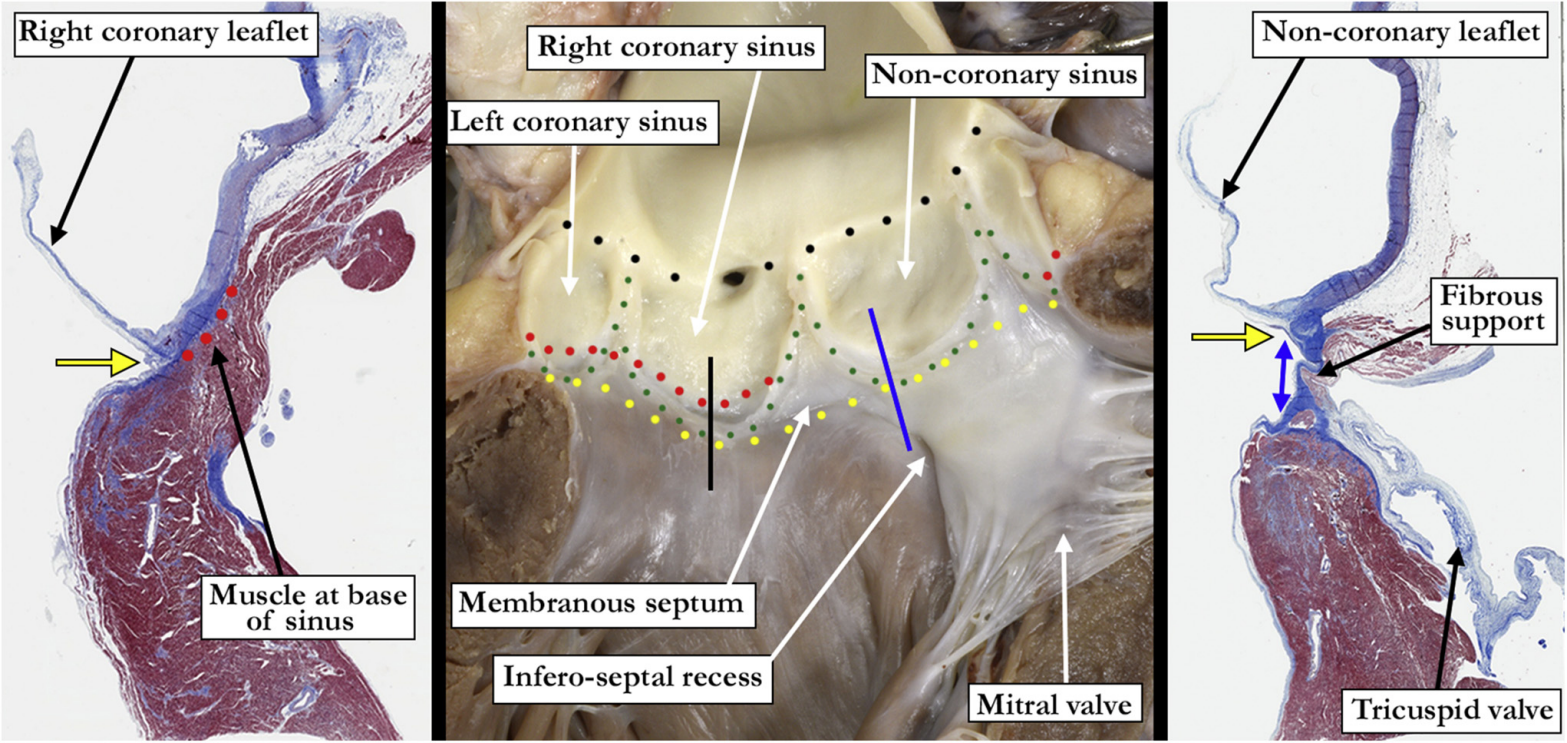
In the *middle panel*, the *yellow dots* mark the **virtual basal ring**, whereas the *red dots* mark the **anatomic ventriculo-aortic junction** with myocardium incorporated into the base of the right and left coronary aortic sinuses. Note the fibrous continuity between the aortic and mitral valves where the muscular support is discontinuous. The *green dots* mark the outline of the aortic valvar sinuses and mark the **hemodynamic ventriculo-aortic ventricular junction**. The *black dots* mark the **sinotubular junction**. The *black line* in the *middle panel* marks the plane of the microscopic section to the left of the *middle panel* showing the muscular support (*red dots*) beneath the right coronary leaflet. The *blue line* marks the plane of section corresponding to the microscopic section in the *right-hand panel* and demonstrates the fibrous support (*double-headed blue arrow*) beneath the noncoronary aortic valvar sinus. In both the *left* and *right-hand panels*, the *yellow arrow* marks the virtual basal ring.

Problems also exist regarding the “annulus.” The consensus, in keeping with the German surgeons, agree this should be represented by the virtual basal ring.^3^ It is a mistake, however, to correlate the virtual basal ring of the aortic root with the ventriculo-aortic junction. Such a discrete junction exists only in the sinuses supporting the coronary arteries (Figure 1).^4^^,^^5^

Only by using an accurate and descriptive account of the normal anatomy of the aortic root will it be possible to achieve the hoped-for consensus.
